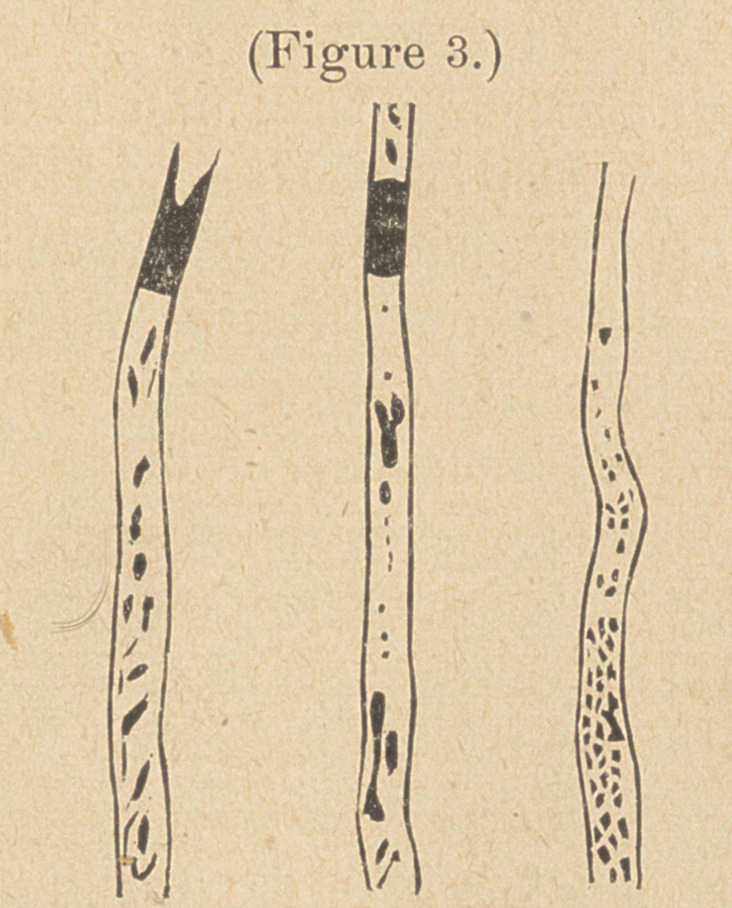# Dental Caries

**Published:** 1883-06

**Authors:** W. D. Miller

**Affiliations:** Berlin


### Independent Practitioner.

VOL. IV. JUNE, 1883. No. 6.

### Original Communications.

### FURTHER CONTRIBUTIONS ON THE SUBJECT OF DENTAL CARIES.

BY W. D. MILLER, BERLIN.

### I. CARIES OF ENAMEL.

Up to the present time, investigations concerning the parasitic nature of dental caries have been confined chiefly, we may almost say exclusively, to a study of its phenomena as seen in connection with carious dentine; but as at least nine-tenths of all the cases of caries which come under our treatment necessarily begin with the enamel, the value of careful observations on the caries of that tissue cannot well be overestimated.

The difficulties attending the preparation of slides of carious enamel are vastly greater than in those of dentine, because in the former the tissue becomes so frail that it falls to pieces during the process of grinding, while the method of treating carious enamel with acids and making razor sections, would hardly be adapted to a study either of the chemical or parasitical character of the carious process.

I have nevertheless succeeded in obtaining one hundred and fifty microscopic preparations of carious enamel, from over one hundred and twenty-five different teeth. These sections were all stained with some aniline dye, and mounted in Canada balsam. Neither enamel nor dentine in a healthy state can, (with the exception mentioned under V) be stained with any of the aniline dyes of which I have made use. On the other hand, both enamel and dentine which have been acted upon by acids, may be readily colored.

This fact often furnishes a very valuable, and sometimes the only indication as to whether any change in the structure or composition of the enamel has taken place.

In the beginning of the carious process we find on the surface of the enamel a slight depression, or concavity, which may or may not contain Leptothrix buccalis in considerable masses. The border of the enamel will be slightly tinted with the coloring matter used in staining; beyond this, to a depth of perhaps fifty micrometers, the enamel will almost always be very perceptibly discolored, as though acted upon by some agent producing effects undistinguishable from those of acids.

In such preparations we must search a long while before we find anything which in any degree justifies the conclusion that the caries is entirely and solely dependent upon the presence of fungi.

In one case, where a considerable portion of the whole periphery seemed to be more or less carious, appearing in various places uneven, and discolored by the dye which had been absorbed, I saw some of the spaces between the enamel prisms apparently distended and filled with something which looked like micrococci. As this something had not been stained at all, the case was rather doubtful.

In another instance, where the enamel was discolored to the depth of about one hundred and fifty micrometers in such a manner as to leave no doubt that a change had taken place, I saw very delicate funnel-shaped excavations extending into the enamel for about ten to twenty micrometers, and apparently containing micrococci. With one or two other exceptions of like nature I have not found in any of my preparations of this class a single instance where even the interstices of the enamel prisms had been penetrated by micro-organisms.

In a second class of preparations, we find the enamel entirely destroyed for a certain space, and the caries encroaching upon the dentine.

(Figure 1.)

Figure 1 may serve to illustrate the manner in which the carious tissue is distributed in such cases.

As soon as the enamel is broken through, the caries extends rapidly in a direction at right angles to the dentinal tubules, as well as parallel with them. Here is a very marked distinction between the action of the agent which produces the softening of the dentine and that of the fungi; for while the former appears to advance with about the same rapidity in all directions, the latter travel very slowly in any course excepting one parallel with the tubules, since they can escape from one tubule to another only through the very narrow and tortuous branches of the tubuli.

This conclusion is fully confirmed by an examination of instances like that cited above, where that part of the softened dentine which is still protected by enamel will be found to contain very few fungi, or none at all.

The walls of enamel on each side of the cavity frequently show a marked discoloration (brownish yellow) extending to a depth varying from a few micrometers to one-fourth of a millimeter ; in other cases an equally broad zone is seen to be distinctly stained by the dye used in preparing the specimen. In such instances the paucity of the micro-organisms is often so great that one must search very carefully to find even a few lying along the margin, or in the fissures of the enamel. Within the enamel itself they do not appear to be present, and a regular advance of the invading organisms beyond or up to the boundary between normal and diseased enamel, seems to be entirely out of the question.

As one result of my study of caries of the enamel, I have been led to the belief that after the enamel has once been broken through and a cavity of decay formed, the destruction of the remaining walls of enamel takes place chiefly from within outwards, rather than from without inwards.

This, which we may call the internal caries of enamel, is somewhat less difficult of examination than the caries of the external surface.

Take for example a molar tooth, extensively decayed on the grinding surface; slightly enlarge the opening in the enamel and it will be found that the bond of union between dentine and enamel has in some places been ruptured. Remove as much as possible of the softened dentine with one stroke of a spoon-shaped excavator, and the surface of the enamel will, to a greater or less extent (depending upon how far the caries has progressed), be seen to be covered with a layer of white, amorphous powder, exactly resembling, both macro- and microscopically, that which is found on the surface of a piece of enamel which has been for some time immersed in acid, or in a mixture of saliva and bread.

Examined microscopically it will be found to consist of enamel prisms either single or in bundles, of from ten to one hundred and fifty micrometres in length. This powder is sometimes half a millimeter in thickness. Remove the surface of the outer layer, repeatedly purifying the instrument by fire and using the most scrupulous care to avoid bringing any kind of impurity in contact with the deeper parts; take then a portion of the powder lying on the border of the healthy enamel, stain and mount it in Canada balsam and you will be astonished to find that there is not only an almost complete absence of micro-organisms where you might have expected to see them in great numbers, but that the enamel also is entirely free from any signs of infection, either between or within the prisms.

In one preparation, by dilligently searching for three minutes, I found one bacillus and two bacteria; in another I hunted for five minutes before I found a single organism. Microscopic sections ground from enamel decaying on its inner surface, frequently show by the readiness with which they take up the dye, that the enamel, to the extent of one-half m. m. or more, has undergone a softening process. But we look in vain for a corresponding invasion of the fungi. The dental fibrils on entering the enamel often become very much expanded, forming oval or spindle-shaped excavations in the substance of the enamel. If the caries of enamel proceeds from within, these cavities readily become filled with fungi, and the zealous seeker after these organisms would no doubt at once jump to the conclusion that they had eaten a hole in the enamel.

I have one preparation of carious enamel in which a very limited number of micrococci appear to have worked their way between the enamel prisms along the course of the enamel fibres, a fact which, however, signifies very little, as I have shown in a previous number of this journal. As a summing up of what has been said, I will state that from a careful study of over one hundred and fifty preparations I am driven to the conclusion that there is as yet no sufficient ground for the assumption that micro-organisms play any more than an unimportant part in the caries of enamel.

### II. CARIES OF CEMENT.

The comparative infrequency of caries of cement compared with that of dentine or enamel, the consequent difficulty of obtaining suitable material for examination, the fact that normal as well as diseased cement becomes to some extent tinged by the aniline dyes, and the absence of any characteristic form in which caries of cement presents itself, combine to make this tissue far more difficult of examination than either the enamel or dentine.

Although I have more than thirty specimens of caries cementum, I have not been able to arrive at an opinion sufficiently conclusive for presentation here. I therefore leave this subject for the present, with the remark that I am not now prepared to present any proofs in favor of a purely parasitic caries of cement.

### III. CARIES OF DENTINE AT NECK OF TOOTH.

The outermost layer of dentine at the neck of the tooth is either normally without tubuli, or they are so fine as not to furnish so easy an entrance to micro-organisms as the tubuli of dentine in general. We therefore find in this region caries manifesting itself in a form somewhat different from that presented by dentine in other positions. It is, moreover, here alone that we may readily obtain specimens of caries of dentine in its incipient stages.

A specimen of this kind, examined under the microscope, usually shows on the outer border a zone consisting of indistinguishable masses of fungi, from which project numerous threads of Leptothrix buccalis. Below this comes a zone of infected dentine, which is seen to be traversed by numerous triangular cracks or fissures, having their bases at the periphery, and apices at some point beneath the surface of the dentine ; in other words, the section appears as though it had been notched by a triangular file. See Figure 2.

(Figure 2.)

These notches are almost invariably found to be filled with fungi, chiefly micrococci. Whether these fissures were produced by the fungi, or whether they resulted from a contraction of the outer layers of the softened dentine, or from some other cause, and afterwards became filled with the fungi, remains to be determined.

Below this zone of what we may call infected dentine, comes a zone of softened, non-infected dentine ; sometimes this is of considerable depth, while in other instances the fissures appear to extend almost if not quite up to the boundary of the normal dentine. In some cases the transition from the softened to the normal dentine is so gradual that it is very difficult to say just where the boundary lies. In a great many, however, it is so abrupt that one may mark the limit with the greatest ease, a zone of deeply stained dentine being immediately followed by one of perfectly colorless tissue, so that one may draw a curved line through the preparation and say : on one side is normal, on the other softened dentine.

This applies not only to that at the neck of a tooth, but to dentine in general, and is a fact hard to reconcile with the germ theory of caries, since in all the preparations which I have examined with reference to this question I have met with very few cases where the boundary line between the infected and non-infected parts is not of the most tortuous and angular nature conceivable.

### IV. CARIES OF DENTINE.

To what I have already written upon this subject in the January number of the *Dental Cosmos* and in the May number of the INDEPENDENT PRACTITIONER, the following may be added: I have made some hundreds of sections of carious dentine, and over two hundred I have treated with various reagents and mounted in Canada balsam. These preparations (unless made from dentine in the last stage of decay) invariably show tracts varying from a small fraction up to one-half of the whole section, which are almost, and sometimes completely, free from micro-organisms. Viewed in this light alone, the idea that softening of the dentine is produced by fungi penetrating the normal dentine and first consuming the organic part, appears to be doubly wrong ; first, because the fungi which should have consumed the organic portion are not there, and second, because the organic matter which should have been consumed is still present, or at most, has suffered less than the inorganic portion.

The same fact may be exemplified in the following manner: Thoroughly cleanse the cavity of a freshly extracted carious tooth in which the pulp is not exposed, remove the softened dentine, repeatedly changing the instrument used for one purified in the flame of a spirit lamp or bunsen-burner, until you have come to the boundary between soft and normal dentine. Then with a spoon-shaped excavator remove a quantity of fine shavings or scrapings of this dentine just on the border, stain and mount in Canada balsam. If the operation has been carefully and cleanly performed but very seldom will any of these shavings contain fungi.

A question in practice is suggested by the following case: A patient comes with a tooth containing a large amalgam or gold filling, made five, ten or more years ago. The filling was inserted over a living pulp, which afterward died, or as frequently happens in Germany, over a dead pulp. An abscess followed and a fistula was formed which has been active at intervals throughout the whole period of ten or more years. During all this time there was probably not a moment when a microscopic examination of the contents of the root-canals would not have revealed great numbers of fungi under conditions of moisture and temperature very favorable to their development. When, therefore, we remove the filling and find no signs of caries in the root, we wonder what those organisms have been doing all these years if, as it is asserted, they are capable of devastating whole rows of teeth, and undermining the best made fillings in the space of a few months. Evidently there is a limit, and we may say with confidence that not all fungi are sufficient to destroy teeth. This is not an imaginary case, but one which I have met with time and again, and one which I think every practitioner must now and then encounter.

### V. PARASITES IN NON-CARIOUS TEETH.

Under this heading I wish to call attention to certain forms met with in the tubuli of perfectly healthy dentine, which may lead us to diagnose fungi when there may be none present. Examining a section of a molar tooth in which the cavity of decay was separated from the pulp by a layer of dentine one-half a c. m. thick, I found on the side of the pulp cavity farthest from the decay, within the dentinal tubuli, something which presented every appearance of micrococci, bacteria and bacilli. Had these tubules been on the side of the pulp cavity presenting the decay, I should not have hesitated a moment to put them down as such. Under higher powers, however, (Zeiss 1-18 oil), the micrococci appeared to have an uneven, irregular contour, while the bacteria and bacilli instead of having roundish ends, sometimes appeared pointed, or as if cut across diagonally, and in some instances I saw shapes which did not correspond with any form of fungus that I have ever seen. Figure 3 roughly illustrates some of these forms.

(Figure 3.)

The tooth from which the section was taken contained a living, healthy pulp. The part of the section containing the fungus-like forms was slightly stained by the dye, the intensity of the color being greatest near the pulp cavity, and extending in some places quite to the periphery of the dentine.

Since that time, in nearly every section of sound dentine, from perfectly sound teeth which I have examined with reference to this question, I have found similar figures, especially the coccus-like form. If these are really fungi, we need no farther proof of their harmlessness than the fact that such sections show no evidence of caries whatever. If, on the other hand, they are not fungi, we are in continual danger of setting down as such, things which are of an entirely different nature.

H. Morgenstern, dentist, of Berlin, described at the last meeting of the Central Verein deutscher Zahnarzte, certain forms which he had found in senile teeth, or such as had become loose from absorption of the alveolus. He believes that he has found either fungi or algæ in such teeth. Whether these are the same as the forms above described, I cannot say. It is significant that he finds them in that class of teeth which is least of all subject to caries.

The case teaches us that it may not be allowable to set down as fungus every little round or oblong thing which we see in a slide.

The points in this communication to which particular attention is directed may be summed up as follows:

(1.) Aniline dyes react upon enamel which is attacked by caries in the same manner as upon enamel softened by acid. A participation of fungi in the first stage of the carious process is not observable, although a slight invasion of the diseased enamel appears in some instances to have taken place.

(2.) Caries of enamel, advancing from the inner surface, reduces the tissue to a fine white powder; sections of such enamel usually show micro-organisms only in the parts where the tissue is completely broken down, while with a proper amount of cleanliness, considerable quantities of the powder may be obtained which is completely free from fungi.

(3.) Softened dentine taken from the surface of the normal dentine appears as a rule quite free from infection.

(4.) The softening of the dentine advances with almost equal rapidity in all directions, while the invasion of fungi goes on much more rapidly in the direction of the canaliculi.

(5.) The boundary between the normal and softened portions of dentine is often perfectly distinct and sharp; it is impossible, on the other hand, to draw the boundary line between the infected and non-infected portions.

(6). The mere presence in the canaliculi of something which resembles, or even actually is, a fungus, is not proof that the thing in question is a cause of caries.

In conclusion, it may be said that the germ theory as tested by the microscope, apparently falls short of furnishing complete explanation of the phenomena of dental caries; that fungi, even when reinforced by acids, do not in all cases prove themselves sufficient for tooth disorganization.

There appears, in many cases at least, to be some other element at work. Investigations in the direction indicated by Prof. Pierce in the *Dental Cosmos* for March, or by Prof. Abbott, ought to throw some light upon this point.

## Figures and Tables

**Figure 1. f1:**
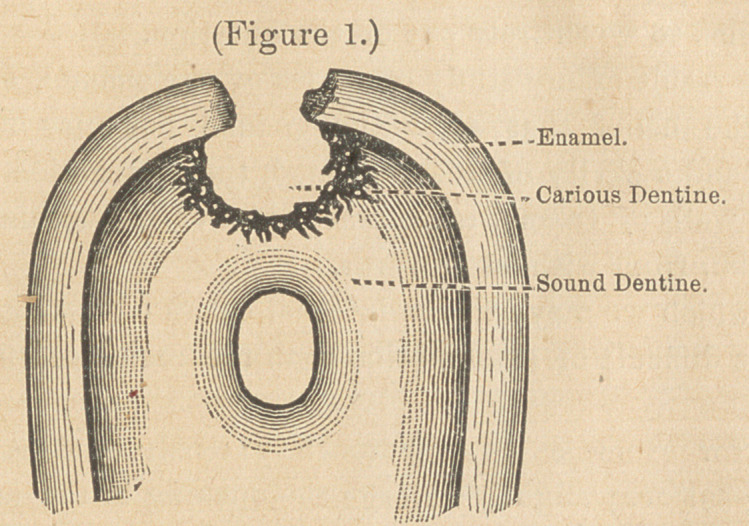


**Figure 2. f2:**
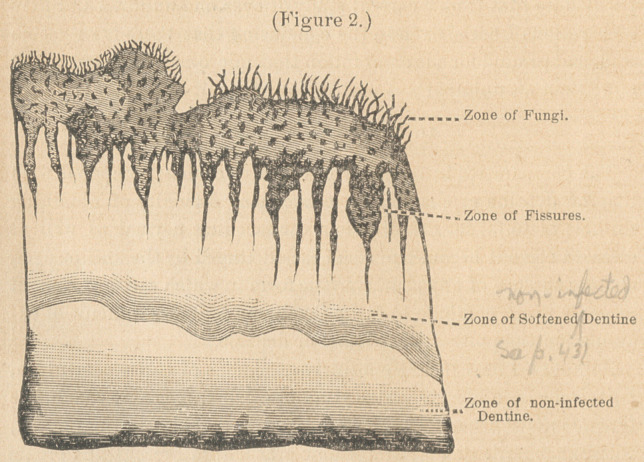


**Figure 3. f3:**